# Effects of Music Genres Reflecting Maternal Listening Preferences During Pregnancy on Distress Markers in Italian Preterm Infants

**DOI:** 10.3390/children13060771

**Published:** 2026-06-02

**Authors:** Barbara Sgobbi, Lorenzo Antichi, Maria Elena Bolis, Laura Morlacchi, Daniele Donati, Ilia Bresesti, Massimo Agosti

**Affiliations:** 1 Neonatal Intensive Care Unit, Department of Women and Child, Del Ponte Hospital, 21100 Varese, Italy; mariaelena.bolis@uninsubria.it (M.E.B.); laura.morlacchi@asst-settelaghi.it (L.M.); ilia.bresesti@uninsubria.it (I.B.); massimo.agosti@uninsubria.it (M.A.); 2 AUOAL Hospital, 15121 Alessandria, Italy; 3 Conservatory “Vivaldi”, 15121 Alessandria, Italy; 4 Humane Technology Lab, Università Cattolica del Sacro Cuore, 20123 Milan, Italy; 5 Department of Medicine and Surgery, University of Insubria, 21100 Varese, Italy; daniele.donati@uninsubria.it

**Keywords:** receptive music therapy, pop/rock genres, prenatal music exposure, NICU, preterm infants

## Abstract

**Highlights:**

**What are the main findings?**

- Classical music (Mozart) was associated with significantly higher peripheral oxygen saturation (SpO_2_) than the no-music condition at three of the five time points; the effect remained significant after correction for multiple comparisons, with medium-to-large effect sizes. No comparable effect emerged for soft pop or soft rock music.
- Music genre was not associated with the LF/HF ratio derived from heart rate variability (HRV) analysis, indicating no measurable genre-specific effect on autonomic distress.

**What are the implications of the main findings?**

- A structured, acoustically monitored receptive music intervention can be delivered to preterm infants in the NICU without signs of overstimulation; the classical-music signal on oxygenation warrants confirmation in larger prospective trials.
- SpO_2_ appears more sensitive than HRV-based measures in detecting the physiological responses of preterm infants to music interventions.

**Abstract:**

**Objective:** This pilot study aimed to explore how a receptive music intervention, based on musical genres reflecting maternal listening preferences during pregnancy, affects distress levels in Italian preterm infants. Specifically, it investigated the effects of soft pop/rock music, compared with classical music, on infants’ LF/HF ratio (derived from heart rate variability [HRV]) and peripheral oxygen saturation (SpO_2_), which were used as physiological markers of distress. **Method:** This retrospective observational pilot study analyzed clinical data routinely collected between May 2014 and January 2015 from 27 preterm infants (gestational age 23–32 weeks; birth weight < 1500 g) who received receptive music therapy as part of standard family-centered care in the NICU. Maternal listening preferences during pregnancy were assessed in 30 mothers via an ad hoc questionnaire; a content analysis identified, at the group level, the three most frequently reported artists (i.e., Jovanotti, Vasco Rossi, and W. A. Mozart), which were used to create three standardized playlists. According to the internal clinical procedure, each infant underwent four sessions on consecutive days: a no-music condition on Day 1, followed by the three music conditions on Days 2–4 in randomized order. The LF/HF ratio and SpO_2_ were measured at five time points per session (one pre-test, three intra-session time points, and one post-test). Wilcoxon signed-rank tests were used to compare conditions and time points, with effect sizes and a Benjamini–Hochberg (FDR) correction for multiple comparisons. **Results:** The LF/HF ratio did not differ significantly across music conditions or relative to the no-music condition. SpO_2_ was higher during the Mozart condition than during the no-music condition at three of the five time points; this association remained significant after FDR correction, with medium-to-large effect sizes. No effect was observed for the soft pop/rock conditions on physiological indexes. **Conclusions:** Receptive music therapy based on maternal listening during pregnancy was not associated with changes in the LF/HF ratio. The Mozart condition was associated with higher SpO_2_ than the no-music condition. Given the small sample, the single-center setting, and the retrospective observational design, these findings are preliminary and require confirmation in larger, adequately powered prospective trials. Future studies should also examine the specific musical features (e.g., tempo, harmonic structure, voice timbre) that may drive these physiological responses.

## 1. Introduction

In recent decades, technological progress in neonatal intensive care has substantially reduced neonatal mortality and enabled the survival of infants born at progressively lower gestational ages. This success has shifted attention toward the quality of survival and the long-term developmental outcomes of preterm infants. Specifically, preterm birth represents a premature transition from the protected intrauterine environment to the NICU, where the infant, whose vascular and neurological systems are immature, is exposed to invasive procedures and intense visual and auditory stimulation [1,2]. These environmental and procedural stressors can impair oxygenation, blood flow, heart rate, and behavioral regulation. Preterm birth is also a stressful and potentially traumatic experience for parents, explaining why developmental and family-centered interventions have become an integral component of NICU care [3,4]. Within this framework, the present study examines whether receptive music therapy, based on musical genres that reflect maternal prenatal listening, can support the physiological regulation of preterm infants. Recently, music and music therapy have been increasingly introduced into NICUs as part of family-centered developmental care [5,6]. A growing body of evidence indicates that music can support preterm infants’ physiological and behavioral self-regulation by improving vital sign stability, feeding competence, and neurodevelopment [7,8,9,10]. Music therapy interventions are generally classified as active, in which infants and parents produce music with the support of a therapist, or receptive, in which infants listen to recorded or live music. Receptive interventions appear safe, feasible, and effective for reducing stress and supporting neurodevelopment in hospitalized infants, with no adverse effects when protocols are applied correctly [11,12]. Despite this evidence, the use of recorded music in the NICU raises safety concerns, as parents and staff increasingly play recorded music to preterm infants through personal devices, sometimes without clinical supervision. Preterm infants have an immature autonomic nervous system (ANS), characterized by a dominant sympathetic branch and a developmentally delayed parasympathetic branch; stressful stimuli, including inappropriately loud or poorly selected music, may exaggerate the sympathetic stress response [4,13,14]. For this reason, recorded music should be administered by a certified music therapist within a structured intervention protocol that monitors both the acoustic environment and the infant’s physiological responses. A further consideration concerns the relationship between the musical material and the prenatal auditory experience. From the third trimester onward, the fetal auditory system is functional, and the fetus can perceive and learn from auditory stimuli transmitted through the womb. Experimental evidence indicates that a melodic contour repeatedly experienced before birth can elicit measurable cardiac responses in the newborn weeks later, suggesting a form of prenatal auditory memory [15,16,17]. Music reflecting the mother’s listening habits during pregnancy may therefore carry a degree of familiarity for the infant. In the present study, the effects of different music genres delivered through receptive music therapy are compared, with maternal prenatal familiarity serving as a criterion for selecting the stimuli. Receptive music interventions in the NICU have most commonly used classical music or lullabies, and recent studies have compared different classical composers [11,18,19]. To the best of our knowledge, however, no study has compared classical music with soft pop/rock music in preterm infants. This gap is relevant because, in our cultural context, the most frequently listened-to music during pregnancy is from the Italian soft pop/rock repertoire. Based on a questionnaire on maternal prenatal listening, three artists emerged as the most frequently reported across the sample—Jovanotti (soft pop), Vasco Rossi (soft rock), and W. A. Mozart (classical)—and were used to build the playlists compared in this study. Infant distress was operationalized primarily through the LF/HF ratio, a frequency-domain index derived from heart rate variability (HRV) that reflects the balance between sympathetic and parasympathetic autonomic activity, with a higher ratio indicating greater autonomic stress; peripheral oxygen saturation (SpO_2_) was used as a complementary physiological indicator. The LF/HF ratio was chosen because it is more sensitive than heart rate, respiratory rate, or observational parameters in detecting changes in the clinical and autonomic stability of preterm infants [20,21].

### Aim

This pilot study aimed to examine the effect of a receptive music intervention comparing different musical genres (soft pop, soft rock, and classical music selected to reflect maternal prenatal listening) on the physiological distress of Italian preterm infants, operationalized as the LF/HF ratio and SpO_2_. Two hypotheses were formulated, grouped by outcome. Regarding the LF/HF ratio, we hypothesized that, compared with the no-music condition, infants would show a lower LF/HF ratio when listening to (a) soft pop/rock music and (b) classical music. Regarding SpO_2_, we hypothesized that, compared with the no-music condition, infants would show higher SpO_2_ levels when listening to (c) soft pop/rock music and (d) classical music. As a secondary question, we explored whether the effects on the two outcomes differed between soft pop/rock and classical music.

## 2. Materials and Methods

### 2.1. Participants

This single-center, retrospective, observational pilot study was conducted in the NICU of the “F. Del Ponte” Hospital in Varese, Italy.

Inclusion criteria were: (a) gestational age between 23 and 32 weeks; (b) birth weight < 1500 g and appropriate for gestational age (>10th percentile); and (c) sufficient stability of vital signs (i.e., heart rate, blood pressure, body temperature, respiratory rate). Exclusion criteria were: (a) the presence of active infections, genetic abnormalities, major congenital heart malformations, or cerebral abnormalities; (b) apneic episodes in the previous 72 h; and (c) failure to pass the bilateral auditory screening.

Infants were classified a priori into two gestational-age subgroups (23–27 weeks, or more immature; 28–32 weeks, or relatively more mature) to account for the marked differences in autonomic, respiratory, and physiological maturation across this gestational-age range.

### 2.2. Materials

Demographic and Clinical Characteristics. The neonatologist collected data on sex, gestational age, ethnicity, and weight of every preterm infant, along with Apgar scores at 1 and 5 min after birth. Survey on Music Preferences. An ad hoc 10-item questionnaire was created to investigate mothers’ musical preferences and which songs and composers mothers listened to most during pregnancy. Five items were dichotomous (e.g., Did you listen to music for your baby during pregnancy?), one used a Likert scale (e.g., What is your favorite music genre?), and four were open-ended (e.g., Which classical music composer did you listen to during your pregnancy?). The full questionnaire is provided in Appendix A.1.

Distress and Physiological Indices. Distress was operationalized using the LF/HF ratio derived from HRV analysis and peripheral oxygen saturation (SpO_2_), measured at five time points 5 min apart (i.e., pre-test, Phase 1, Phase 2, Phase 3, and post-test). HRV reflects autonomic nervous system fluctuations. It is more sensitive than other markers, such as heart rate, respiratory rate, and observational parameters, for detecting behavioral changes and levels of distress [20,22]. HRV frequency-domain estimates quantify variance in the high-frequency (HF) and low-frequency (LF) regions of the HRV spectrum. Different ANS physiological phenomena influence these regions: the parasympathetic branch influences the HF region, and the sympathetic branch influences the LF region. Distress conditions are associated with increased LF power, reduced HF power, and an increased LF/HF ratio; conversely, a decreased LF/HF ratio reflects relatively greater parasympathetic modulation and lower distress. Accordingly, in this study, a lower LF/HF ratio was interpreted as indicating lower distress [21,22]. HRV was assessed using electrocardiography (ECG). The analog ECG signal from a heart rate monitor (minicardio MC030, Hosand Technologies Srl Via Marradi 1, 20123 Milano, Italy) was fed into a computer running HRV software (Train Me Coach, Hosand Technologies Srl Via Marradi 1, 20123 Milano, Italy) and converted into digital RR-interval values, reflecting the time elapsed between two successive R-waves. The device recorded the ECG signal in mV, detected the R peak, and stored the RR interval with a sampling accuracy of 1000 Hz (1 Hz = 1 cycle/s). Sympathetic influences appear in the low-frequency (LF) band (<0.15 Hz), and parasympathetic influences predominantly in the high-frequency (HF) band (0.15–0.40 Hz).

Finally, the SpO_2_ level (i.e., the percentage of oxygen-saturated hemoglobin in peripheral blood) was measured using an OXY200 pulse oximeter (model 110280-OXY200) connected to the monitor (Philips MedizinSysteme Germany IntelliVue MX800 iPC), with the sensors attached to the infants’ feet. All measurements were performed using the same device, which was connected to the incubator (Dräger Babyleo TN500).

### 2.3. Procedure

This retrospective observational pilot study analyzed clinical data routinely collected between May 2014 and January 2015 from preterm infants who received receptive music therapy as part of standard family-centered care in our NICU. Receptive music therapy has been part of routine care in this NICU at the “F. Del Ponte” Hospital since 2013, according to an internal clinical procedure. The present study did not modify that procedure but retrospectively analyzed the physiological data routinely recorded during it. The study documentation was reviewed by the Ethics Committee of Insubria (ref. 51/2019, 7 May 2019), which classified it as a retrospective observational study and issued a formal acknowledgment. Parents had provided written informed consent for the music therapy and for the anonymous scientific use of the clinical data.

**Figure 1:**
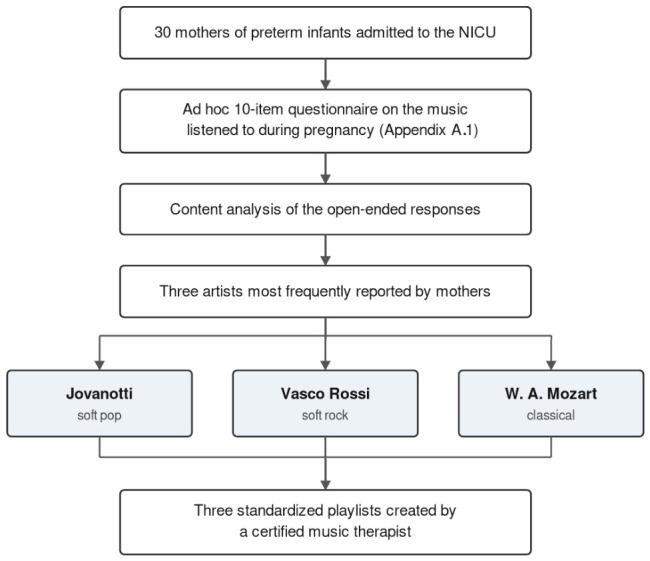
Selection of the musical material. Flowchart of the music selection process. 30 mothers of preterm infants completed an ad hoc questionnaire about the music they listened to during pregnancy. A content analysis of the open-ended responses identified, at the group level, the three most frequently reported artists (Jovanotti, soft pop; Vasco Rossi, soft rock; W. A. Mozart, classical), which were used to build three standardized playlists.

Before the analyzed sessions began, 30 mothers of preterm infants admitted to the NICU completed an ad hoc 10-item questionnaire investigating the music they had listened to during pregnancy (see Appendix A.1). The questionnaire explored the musical tastes of both parents; for the present analysis, maternal listening history served as the primary prenatal reference. A content analysis of the open-ended responses was conducted to identify the artists most frequently reported by the group of mothers. The three most frequently mentioned names were Jovanotti (soft pop), Vasco Rossi (soft rock), and W. A. Mozart (classical); the frequency of each artist or composer is presented in Supplementary Table S5 and Figure S1. On this basis, a certified music therapist created three standardized playlists: (a) a soft-pop playlist including three songs by Jovanotti; (b) a soft-rock playlist including three songs by Vasco Rossi; and (c) a classical playlist including the Mozart compositions KV 299 (Andantino) and KV 622 (Adagio). As with the pop and rock songs, the specific Mozart compositions were those most frequently reported by the mothers in the questionnaire. The overall selection process is summarized in Figure 1. The three repertoires differed in musical structure: Jovanotti’s songs are sustained, regular, and repetitive; Vasco Rossi’s songs are more varied, with tempo changes and rhythmic-harmonic variation in the refrain; and Mozart’s compositions follow a simple, repetitive classical structure with minor rhythmic and melodic variation (see Supplementary Table S6 and Appendix A.2 for a detailed analysis of the songs’ musical characteristics).

**Figure 2:**
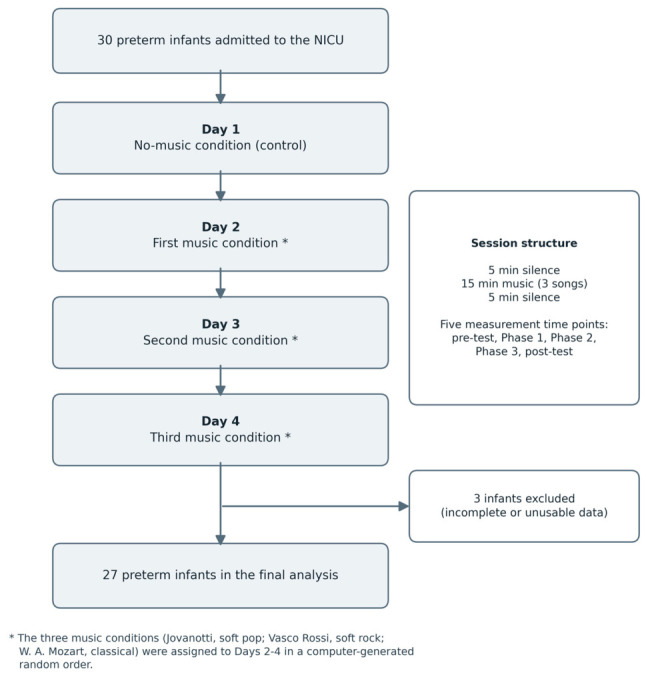
Study procedure. Flowchart of the study procedure. Each infant underwent four conditions on separate days: a no-music (control) condition and the Jovanotti, Vasco Rossi, and Mozart conditions, the latter three administered in randomized order. Each session comprised 5 min of silence, 15 min of music, and 5 min of silence, and physiological parameters were recorded at five time points.

According to the internal clinical procedure, each infant underwent four conditions on four consecutive days: a no-music condition (control, with physiological recording only) on Day 1, followed by the Jovanotti, Vasco Rossi, and Mozart conditions on Days 2–4. The order of the three music playlists across Days 2–4 was determined by a computer-generated random sequence, prepared in advance of the sessions as part of the clinical procedure (see Figure 2 for the procedure flowchart). For all infants, the four sessions began once the infant had reached 15 days of postnatal life, with similar postnatal ages across infants. The corresponding postmenstrual age at the start of the sessions, computed as gestational age plus chronological age, was 30 weeks (range: 25–34 weeks).

Music was administered through speakers placed in the incubator, where each infant lay. The methodology and acoustic monitoring followed the Lubetzky criteria [19]. Two types of neonatal incubators were used: an older model without an integrated audio system and a newer model (Dräger Babyleo TN500) with a built-in loudspeaker positioned 30 cm from the newborn’s ear, whose volume was adjusted according to the infant’s behavior, HRV, and SpO_2_. For the older incubator, an external speaker was placed to reproduce the same acoustic conditions. Music was played from an iPod, and the loudspeaker output was calibrated to meet the recommendations of the American Academy of Pediatrics [23]. The sound level was monitored throughout each session with a calibrated professional sound-level meter (Trotec SL400) placed inside the incubator. The literature reports that, in NICUs with older technology and open-space layouts, ambient noise inside incubators with CPAP ventilation active ranges from a mean of about 70 dBA to peaks of about 80 dBA, well above the internationally recommended thresholds of a mean below 50 dBA and peaks below 70 dBA [24,25]. In the present study, conducted in family single rooms with modern incubators, the external background sound level had a mean below 55 dBA and peaks of at most 70 dBA. During the music sessions, with the safety measures in place, the sound level measured inside the incubator, near the infant’s ear at 30 cm, remained below 50 dBA, with background-noise peaks of at most 65 dBA, even with ventilators active. To minimize background noise, monitors and alarms were set to visual signals only, all doors were closed, and staff were asked not to speak during the music. Each music therapy session lasted 25 min: 5 min of silence before the music, 15 min of listening, and 5 min of silence afterward; the 15 min duration was based on the literature [10]. Sessions were always administered between 12:00 and 13:00, about two hours after the morning care routine (bathing, cleaning, etc.) and before mothers met their infants. Sessions were performed only when infants were clinically stable, as judged by the neonatologist, at preset quiet times of the day, after feeding and routine care. Throughout each session, neonatal vital signs were continuously monitored, and the neonatologist observed the infant to detect any changes indicative of overstimulation or instability (in which case the music would be stopped). No painful events or other clinical difficulties were detected during these periods. An electrocardiogram (ECG) was recorded during each session to derive the LF/HF ratio from HRV analysis, and SpO_2_ was measured with a pulse oximeter.

### 2.4. Data Analysis

Descriptive statistics were calculated using the Statistical Package for the Social Sciences (SPSS, version 25). Little’s MCAR test was performed to assess whether the missing data were missing completely at random. Independent-samples *t*-tests were run to investigate differences in birth weight and Apgar scores at 1 and 5 min between the two gestational-age subgroups (23–27 weeks vs. 28–32 weeks), with Levene’s test used to assess homogeneity of variances for each comparison. The LF/HF ratio and SpO_2_ were compared both between conditions and within each condition across time points. Because all comparisons were within-subject (each infant underwent every condition on separate days), the sample size was small, and the distributions of the two outcomes were non-normal, the non-parametric Wilcoxon signed-rank test was used for all paired comparisons. For each comparison, an effect size was computed as r = |Z|/√N, where N is the number of pairs; following Cohen’s conventions, values of r of approximately 0.10, 0.30, and 0.50 were interpreted as small, medium, and large, respectively [26]. Moreover, *p*-values were corrected using the Benjamini–Hochberg false discovery rate (FDR) procedure to account for multiple testing. Comparisons were grouped into families, and the correction was applied separately within each family. Four families corresponded to the pre-specified hypotheses (a–d): pop/rock versus no-music and classical versus no-music for the LF/HF ratio; and pop/rock versus no-music and classical versus no-music for SpO_2_. The remaining comparisons were exploratory and were corrected within separate families: the direct comparisons between the two music genres (one family per outcome) and the within-condition comparisons across time points (one family per condition and outcome). Both uncorrected and FDR-corrected *p* values are reported. Missing data were handled with pairwise deletion.

## 3. Results

Thirty Italian preterm infants were recruited. Three infants were excluded because of the complete absence of data and unusable HRV recordings. Moreover, physiological data were missing for some enrolled participants (ranging from 1 to 4 observations per variable). Little’s MCAR test was significant, indicating a pattern that was likely not completely random (χ^2^ (460) = 220,615.34, *p* < 0.001). This pattern could be due to the infants’ involuntary movements detaching the electrodes placed on their bodies, preventing accurate HRV detection.

The final sample comprised 27 preterm infants: 13 in the 23–27-week subgroup (48.1%) and 14 in the 28–32-week subgroup (51.9%). There were 19 males (70.4%) and 8 females (29.6%). The median gestational age was 28 weeks [IQR 26–30]. The median birth weight was 1040 g [IQR 870–1280]. The median Apgar scores were 6 [IQR 5–7] at 1 min and 8 [IQR 8–8] at 5 min (see Table 1 for the demographic characteristics). Regarding the clinical differences between infants in the 23–27-week and 28–32-week subgroups, Levene’s test showed homogeneity of variances for weight (F = 0.40, *p* = 0.533), Apgar 1 (F = 0.03, *p* = 0.88), and Apgar 5 (F = 3.27, *p* = 0.08). Specifically, infants in the 23–27-week subgroup had a significantly lower weight (t(25) = −5.22, *p* < 0.001, 95% CI [−612.81, −265.83]) and Apgar 1 scores (t(25) = −2.60, *p* = 0.015, 95% CI [−2.38, −0.28]) than infants in the 28–32-week subgroup, whereas the difference in Apgar score at 5 min was not statistically significant (t(25) = −0.80, *p* = 0.43, 95% CI [−0.53, 0.24]). The descriptive statistics (median and interquartile range) of the LF/HF ratio and SpO_2_ for each condition and time point are reported in Table 2.

**Figure 3:**
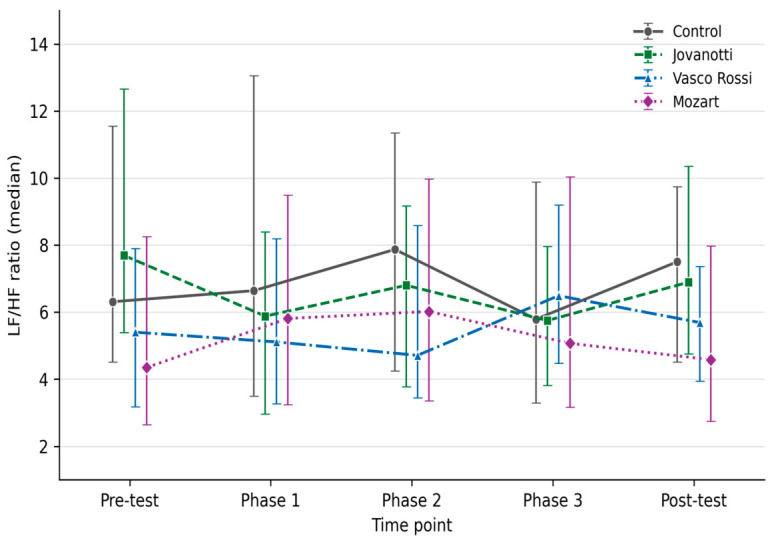
LF/HF ratio by condition across the five time points. Median LF/HF ratio for the four conditions at the pre-test, the three intra-session phases, and the post-test. Error bars represent the interquartile range. Higher LF/HF values indicate greater autonomic stress. Markers are slightly offset horizontally to avoid overlap. LF/HF = low-frequency/high-frequency ratio derived from heart rate variability.

Sub-hypothesis (a), that infants listening to soft pop/rock music would show a lower LF/HF ratio than the no-music condition, was not confirmed. At the pre-test, the Jovanotti condition showed a higher LF/HF ratio than the Vasco Rossi (Z = 2.38, *p* = 0.017, r = 0.48) and the Mozart (Z = 2.38, *p* = 0.018, r = 0.47) conditions, with medium effect sizes; however, these baseline genre-to-genre differences did not survive the FDR correction and are interpreted as pre-existing variability rather than an intervention effect. No significant differences in the LF/HF ratio emerged between the Jovanotti, Vasco Rossi, and control conditions at Phase 1, Phase 2, Phase 3, or the post-test (see Table 3; see also Supplementary Table S1 for the full between-condition Wilcoxon results). Within conditions, the LF/HF ratio did not change significantly across time points in the Vasco Rossi or control conditions. In the Jovanotti condition, the LF/HF ratio was lower at Phase 2 than at the pre-test (Z = −2.02, *p* = 0.043, r = 0.40), but this result did not survive the FDR correction and should not be interpreted as a reliable effect (see Table 4; see also Supplementary Table S4 for the full within-condition Wilcoxon results and Figure 3).

Sub-hypothesis (b), that infants listening to classical music would show a lower LF/HF ratio than the no-music condition, was not confirmed. There were no significant differences in the LF/HF ratio between the Mozart and control conditions at any phase, and no significant differences between the classical and the soft pop/rock conditions at any phase (see Table 3 and Figure 3). Within the Mozart condition, the LF/HF ratio did not change significantly across time points (see Table 4; see also Supplementary Table S4 for the full Wilcoxon results).

**Figure 4:**
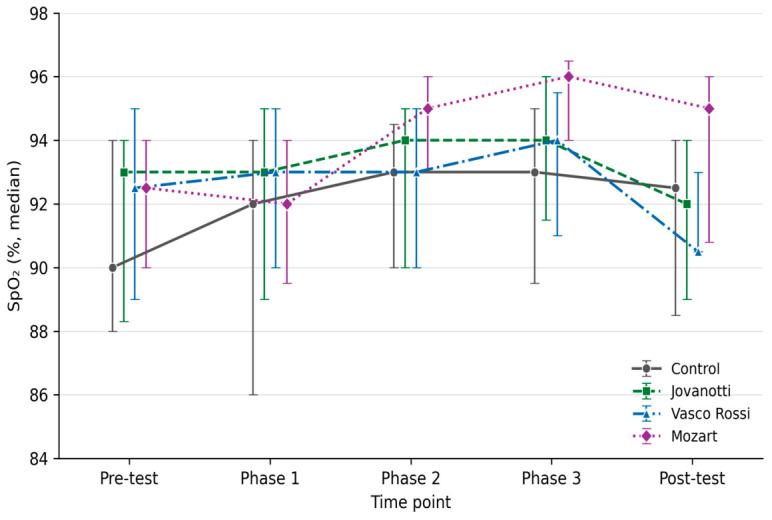
Peripheral Oxygen Saturation by condition across the five time points. Median peripheral oxygen saturation (SpO_2_, %) for the four conditions across the five time points. Error bars represent the interquartile range. Markers are slightly offset horizontally to avoid overlap.

Sub-hypothesis (c), that infants listening to soft pop/rock music would show higher SpO_2_ levels than the no-music condition, was not confirmed. There were no significant differences in SpO_2_ between the Jovanotti or Vasco Rossi conditions and the control condition at any phase (see Table 3 and Figure 4; see also Supplementary Table S2 for the full between-condition Wilcoxon results). The exploratory within-condition analyses showed no significant change in SpO_2_ over time in the control condition. In the Jovanotti condition, SpO_2_ increased over time: it was higher at Phase 3 than at the pre-test (Z = 3.11, *p* = 0.002, r = 0.62), at Phase 1 (Z = 2.18, *p* = 0.029, r = 0.44), and at the post-test (Z = 2.69, *p* = 0.007, r = 0.54), and higher at Phase 2 than at the pre-test (Z = 2.13, *p* = 0.033, r = 0.43). After FDR correction, two of these increases remained significant: the higher SpO_2_ at Phase 3 compared with the pre-test (p_FDR = 0.020) and compared with the post-test (p_FDR = 0.035). The increase at Phase 3 compared with Phase 1, and the increase at Phase 2 compared with the pre-test, did not survive the correction. In the Vasco Rossi condition, SpO_2_ was higher at Phase 3 than at the post-test (Z = 2.42, *p* = 0.015, r = 0.48), but this result did not survive the FDR correction (see Table 4; see also Supplementary Table S3 for the full within-condition Wilcoxon results and Figure 4).

Sub-hypothesis (d), that infants listening to classical music would show higher SpO_2_ levels than the no-music condition, was confirmed (see Table 3, Supplementary Table S3, and Figure 4). SpO_2_ did not differ significantly between the Mozart and control conditions at the pre-test or at Phase 1. Still, it was higher in the Mozart condition at Phase 2 (Z = 2.31, *p* = 0.021, r = 0.45), Phase 3 (Z = 3.11, *p* = 0.002, r = 0.60), and the post-test (Z = 2.73, *p* = 0.006, r = 0.53). These three differences, with medium-to-large effect sizes, all remained significant after FDR correction (p_FDR = 0.035, 0.010, and 0.015). At Phase 2, SpO_2_ in the Mozart condition was also higher than in the Jovanotti (Z = 2.01, *p* = 0.043, r = 0.41) and Vasco Rossi (Z = 2.14, *p* = 0.032, r = 0.43) conditions; these genre-to-genre differences did not survive the FDR correction. Within the Mozart condition, SpO_2_ increased markedly over time: it was higher at Phase 2 than at the pre-test (Z = 3.89, *p* < 0.001, r = 0.75) and Phase 1 (Z = 3.62, *p* < 0.001, r = 0.70); higher at Phase 3 than at the pre-test (Z = 3.71, *p* < 0.001, r = 0.71), Phase 1 (Z = 3.56, *p* < 0.001, r = 0.69), and the post-test (Z = 2.15, *p* = 0.032, r = 0.41); and higher at the post-test than at the pre-test (Z = 2.70, *p* = 0.007, r = 0.52) and Phase 1 (Z = 2.79, *p* = 0.005, r = 0.54). All these within-condition increases, with mostly large effect sizes, remained significant after FDR correction (see Table 4; see also Supplementary Table S3 and Figure 4). 

## 4. Discussion

This pilot study examined how a receptive music intervention, comparing soft pop/rock and classical music genres, selected to reflect maternal prenatal listening, was associated with physiological distress markers in Italian preterm infants. Distress was operationalized through the LF/HF ratio derived from HRV analysis and through peripheral oxygen saturation (SpO_2_).

### 4.1. The LF/HF Ratio

Sub-hypotheses (a) and (b) were not confirmed. Music genre was not associated with the LF/HF ratio, since neither soft pop/rock nor classical music produced a lower LF/HF ratio than the no-music condition at any phase. At the pre-test, the Jovanotti condition showed a higher LF/HF ratio than the Vasco Rossi and Mozart conditions. Still, these differences did not survive the FDR correction and most likely reflect pre-existing variability in baseline distress rather than an effect of the intervention. The single within-condition result, a lower LF/HF ratio at Phase 2 than at the pre-test in the Jovanotti condition, also did not survive the FDR correction and is not interpreted as a reliable effect. Within the Mozart condition, the LF/HF ratio did not change over time.

These results should be interpreted in light of the properties of this index. The LF/HF ratio is a recognized frequency-domain measure of sympathovagal balance. Still, no universally accepted normal range exists for preterm infants, and its values depend on gestational and postnatal age, behavioral state, and recording conditions [20,21,22]. The absence of a measurable genre effect on this marker should therefore be interpreted with caution. One possible interpretation concerns the musical material. The compositions used were structurally richer than the simple, repetitive music typically employed in the NICU. Extremely preterm infants have a markedly immature nervous system and may not be able to process complex melodic structures in the early stages of life [27]. Simpler structures, such as lullabies, may have a stronger effect on distress, as suggested by previous work [7,9,18,28,29,30,31]. Direct comparison with the literature is limited because no previous study has examined the effect of soft pop/rock music on the LF/HF ratio of preterm infants. For classical music, Mikulis et al. [11] reported that a Mozart-based intervention reduced resting energy expenditure, heart rate, respiratory rate, and stress levels; that study did not assess the LF/HF ratio, so a direct comparison is not possible.

### 4.2. Peripheral Oxygen Saturation (SpO_2_)

Sub-hypothesis (c) was not confirmed: SpO_2_ did not differ between the soft pop/rock conditions and the no-music condition at any phase. The exploratory within-condition analyses showed an increase in SpO_2_ over time in the Jovanotti condition; after FDR correction, the increase at Phase 3 relative to the pre-test and post-test remained significant, whereas the other comparisons did not. Sub-hypothesis (d) was confirmed. SpO_2_ was higher in the Mozart condition than in the no-music condition at Phase 2, Phase 3, and the post-test, with medium-to-large effect sizes, and these differences remained significant after FDR correction. The within-condition increase in SpO_2_ during the Mozart sessions was consistent and remained significant after correction, with mostly large effect sizes. This finding is consistent with Shin et al. [32], who reported higher SpO_2_ levels in infants exposed to Mozart than in those receiving standard care. The direct comparisons between the Mozart condition and the soft pop/rock conditions at Phase 2 were nominally significant but did not survive the FDR correction; we therefore do not claim that classical music is superior to soft pop/rock music for SpO_2_.

Furthermore, the LF/HF ratio and SpO_2_ exhibited distinct patterns, and this apparent discrepancy warrants comment. The two indices reflect different physiological systems: the LF/HF ratio indexes autonomic balance, whereas SpO_2_ reflects peripheral oxygenation and respiratory stability. Hence, they are not expected to change in parallel. Indeed, SpO_2_ may respond more readily to a short receptive intervention than the LF/HF ratio, which depends on a still-immature autonomic system. The increase in SpO_2_ should therefore be read as a change in one physiological marker, and not as direct evidence of reduced autonomic distress. This interpretation is further tempered by the number of comparisons performed, which is why the FDR correction was applied.

### 4.3. Clinical Implications

Within a family-centered care framework, these findings suggest that a structured receptive music intervention can be delivered to preterm infants in the NICU without signs of overstimulation. The acoustic environment was monitored throughout the sessions with a professional sound-level meter, and the sound level inside the incubator remained below 50 dBA, within the limits recommended for the NICU. The involvement of a certified music therapist is important because the therapist can monitor the infant’s responses and judge whether recorded music poses a risk. The recent Cochrane review by Haslbeck et al. [33] reported no adverse effects associated with recorded music interventions. Because each condition was administered only once, the present results do not address the effect of repeated exposure. A first exposure to a piece of music may elicit an orienting or arousal response rather than a stable adaptation, and repeated-exposure designs would clarify whether the observed changes in SpO_2_ are sustained.

### 4.4. Strengths

The study has several strengths. To our knowledge, it is the first to investigate maternal prenatal music preferences in relation to the physiological responses of preterm infants, and the first to compare soft pop/rock with classical music in this population. Every infant underwent all conditions, thereby reducing the influence of between-infant variability. The acoustic environment was monitored with a calibrated instrument, in line with NICU sound-safety recommendations. Distress was assessed using objective physiological measures rather than relying solely on behavioral observations. Finally, the intervention is low-cost and non-pharmacological, and is therefore well-suited to developmental care.

### 4.5. Limitations

Nevertheless, this study has several limitations. First, the small sample size limited statistical power and the generalizability of the findings. Second, because the design was retrospective and observational, the results describe associations rather than support causal conclusions. Third, although multiple comparisons were corrected for FDR, the confirmatory findings should still be regarded as preliminary. Fourth, several clinical variables that may influence the LF/HF ratio and SpO_2_ were not systematically recorded, including feeding state, sleep–wake state, respiratory support, ongoing medication, recent painful procedures, and ambient noise. Fifth, missing data were present and were handled using pairwise deletion. Sixth, each condition was administered only once, preventing any assessment of cumulative or repeated-exposure effects. Seventh, the playlists reflected the listening preferences of Italian families, which may limit the generalizability of the findings culturally and musically. In addition, physiological indices were recorded every 5 min; therefore, changes occurring between time points were not captured, and the Wilcoxon signed-rank test could not model the rate of change in the physiological curves. Finally, the spectral content and A-weighted equivalent continuous sound level (LAeq) of the musical stimuli were not computed from the original recordings.

## 5. Conclusions

This pilot study examined whether different music genres, selected to reflect maternal prenatal listening, were associated with physiological markers of distress in preterm infants. Music genre was not associated with the LF/HF ratio, indicating that the intervention did not measurably affect autonomic distress as indexed by this marker. Classical music (Mozart) was associated with higher SpO_2_ than the no-music condition, and this association remained significant after correction for multiple comparisons, with medium-to-large effect sizes. No corresponding effect was found for soft pop/rock music. These findings should be regarded as preliminary and hypothesis-generating. The study was a small, retrospective, observational pilot, so the results describe associations rather than establishing that music reduces distress. The increase in SpO_2_ in the Mozart condition is a promising signal that warrants confirmation. The intervention appeared to be well tolerated when delivered by a certified music therapist within a structured, acoustically monitored procedure. Adequately powered prospective studies are needed to confirm these observations. Future research should use larger samples, examine repeated and longer-term exposure, and consider the songs each mother listened to most during pregnancy, rather than those most common across the group. Other genres, such as rap, could also be examined. It would be informative to analyze the specific acoustic parameters of the stimuli, such as melodic line, harmonic structure, voice timbre, rhythm, tuning, and instrumentation, to identify which features drive physiological responses. Finally, longitudinal modeling approaches, such as linear mixed models, generalized estimating equations, or latent growth curve models, would better capture the rate of change in the physiological curves over time.

## Figures and Tables

**Table 1:** Demographic and clinical characteristics of the preterm infants (N = 27).

Characteristic	Value
Gender, n (%)	
Male	19 (70.4)
Female	8 (29.6)
Gestational age (weeks), median [IQR]	28 [26–30]
23–27 weeks, n (%)	13 (48.1)
28–32 weeks, n (%)	14 (51.9)
Birth weight (g), median [IQR]	1040 [870–1280]
Apgar score at 1 min, median [IQR]	6 [5–7]
Apgar score at 5 min, median [IQR]	8 [8–8]

Note. IQR = interquartile range. Categorical variables are presented as counts and percentages, n (%); continuous variables as the median with the interquartile range [25th–75th percentile].

**Table 2:** Descriptive statistics of the LF/HF ratio and SpO_2_ by condition and time point.

Condition/Time Point	LF/HF Ratio		SpO_2_	
	N	Median [IQR]	N	Median [IQR]
*Control*				
Pre-test	23	6.31 [4.52–11.55]	27	90.0 [88.0–94.0]
Phase 1	24	6.64 [3.49–13.05]	27	92.0 [86.0–94.0]
Phase 2	24	7.87 [4.25–11.35]	27	93.0 [90.0–94.5]
Phase 3	23	5.79 [3.29–9.89]	27	93.0 [89.5–95.0]
Post-test	24	7.50 [4.51–9.74]	27	92.5 [88.5–94.0]
*Jovanotti*				
Pre-test	26	7.69 [5.39–12.66]	25	93.0 [88.3–94.0]
Phase 1	26	5.88 [2.96–8.40]	25	93.0 [89.0–95.0]
Phase 2	26	6.80 [3.77–9.17]	25	94.0 [90.0–95.0]
Phase 3	25	5.75 [3.81–7.96]	25	94.0 [91.5–96.0]
Post-test	26	6.89 [4.76–10.36]	25	92.0 [89.0–94.0]
*Vasco Rossi*				
Pre-test	25	5.40 [3.18–7.90]	25	92.5 [89.0–95.0]
Phase 1	25	5.11 [3.27–8.19]	25	93.0 [90.0–95.0]
Phase 2	25	4.71 [3.45–8.59]	25	93.0 [90.0–95.0]
Phase 3	24	6.49 [4.47–9.20]	25	94.0 [91.0–95.5]
Post-test	25	5.69 [3.94–7.37]	25	90.5 [90.5–93.0]
*Mozart*				
Pre-test	27	4.35 [2.64–8.25]	27	92.5 [90.0–94.0]
Phase 1	27	5.81 [3.24–9.49]	27	92.0 [89.5–94.0]
Phase 2	27	6.02 [3.35–9.97]	27	95.0 [93.0–96.0]
Phase 3	26	5.07 [3.16–10.04]	27	96.0 [94.0–96.5]
Post-test	27	4.58 [2.75–7.97]	27	95.0 [90.8–96.0]

Note. LF/HF = low-frequency/high-frequency ratio derived from heart rate variability; SpO_2_ = peripheral oxygen saturation; IQR = interquartile range. Values are medians with the interquartile range [25th–75th percentile]. N = number of valid (non-missing) cases.

**Table 3:** Wilcoxon signed-rank tests of each music condition versus the no-music condition.

Comparison	LF/HF Ratio				SpO_2_			
	*Z*	*p*	p(FDR)	*r*	*Z*	*p*	p(FDR)	*r*
*Pre-test*								
Jovanotti	−0.80	0.426	0.776	0.17	−0.20	0.843	0.958	0.04
Vasco Rossi	−1.38	0.168	0.776	0.29	−1.45	0.146	0.415	0.29
Mozart	−1.73	0.083	0.208	0.36	−0.59	0.558	0.693	0.11
*Phase 1*								
Jovanotti	−0.61	0.543	0.776	0.13	−0.05	0.958	0.958	0.01
Vasco Rossi	−0.82	0.412	0.776	0.17	−1.39	0.166	0.415	0.28
Mozart	−1.09	0.278	0.463	0.22	−0.40	0.693	0.693	0.08
*Phase 2*								
Jovanotti	−0.70	0.484	0.776	0.15	−0.07	0.943	0.958	0.01
Vasco Rossi	−1.52	0.128	0.776	0.32	−0.17	0.863	0.958	0.03
Mozart	−0.46	0.648	0.783	0.09	−2.31	0.021	**0.035**	0.45
*Phase 3*								
Jovanotti	−0.43	0.664	0.830	0.09	−1.86	0.063	0.415	0.37
Vasco Rossi	−0.30	0.768	0.853	0.07	−1.52	0.128	0.415	0.30
Mozart	−0.28	0.783	0.783	0.06	−3.11	0.002	**0.010**	0.60
*Post-test*								
Jovanotti	−0.03	0.976	0.976	0.01	−0.36	0.716	0.958	0.07
Vasco Rossi	−1.10	0.274	0.776	0.23	−0.67	0.501	0.958	0.13
Mozart	−1.80	0.072	0.208	0.37	−2.73	0.006	**0.015**	0.53

Note. Each row reports the comparison between the indicated music condition and the no-music (control) condition at the same time point. Z = test statistic; *p* = uncorrected significance; p(FDR) = significance after Benjamini–Hochberg false discovery rate correction, applied within each pre-specified hypothesis family; r = effect size, computed as r = |Z|/√N. Following Cohen, r ≈ 0.10, 0.30, and 0.50 indicate small, medium, and large effects. Values of p(FDR) < 0.05 are shown in bold. LF/HF = low-frequency/high-frequency ratio; SpO_2_ = peripheral oxygen saturation. Genre-to-genre comparisons are reported in Supplementary Tables S1 and S2.

**Table 4:** Wilcoxon signed-rank tests of each time point versus the pre-test, within conditions.

Comparison	LF/HF Ratio				SpO_2_			
	*Z*	*p*	p(FDR)	*r*	*Z*	*p*	p(FDR)	*r*
*Control*								
Phase 1	−0.43	0.670	0.838	0.09	−0.50	0.618	0.696	0.10
Phase 2	−0.55	0.584	0.834	0.12	−1.35	0.178	0.580	0.26
Phase 3	−0.93	0.355	0.775	0.20	−0.72	0.475	0.696	0.14
Post-test	−0.73	0.465	0.775	0.15	−0.46	0.647	0.696	0.09
*Jovanotti*								
Phase 1	−1.88	0.060	0.200	0.37	−0.34	0.737	0.819	0.07
Phase 2	−2.02	0.043	0.200	0.40	−2.13	0.033	0.082	0.43
Phase 3	−1.90	0.058	0.200	0.38	−3.11	0.002	**0.020**	0.62
Post-test	−0.72	0.469	0.938	0.14	−0.14	0.891	0.891	0.03
*Vasco Rossi*								
Phase 1	−0.26	0.798	0.841	0.05	−1.06	0.290	0.488	0.21
Phase 2	−0.34	0.737	0.841	0.07	−0.15	0.882	0.896	0.03
Phase 3	−0.71	0.475	0.841	0.14	−1.12	0.263	0.488	0.22
Post-test	−0.35	0.726	0.841	0.07	−0.82	0.414	0.591	0.16
*Mozart*								
Phase 1	−0.79	0.428	0.869	0.15	−0.08	0.939	0.939	0.01
Phase 2	−1.01	0.313	0.869	0.19	−3.89	<0.001	**0.001**	0.75
Phase 3	−0.37	0.713	0.869	0.07	−3.71	<0.001	**0.001**	0.71
Post-test	−0.17	0.869	0.869	0.03	−2.70	0.007	**0.012**	0.52

Note. Each row reports the comparison between the indicated time point and the pre-test within the same condition. Z = test statistic; *p* = uncorrected significance; p(FDR) = significance after Benjamini–Hochberg false discovery rate correction, applied within each condition-by-outcome family; r = effect size, computed as r = |Z|/√N. Following Cohen, r ≈ 0.10, 0.30, and 0.50 indicate small, medium, and large effects. Values of p(FDR) < 0.05 are shown in bold. LF/HF = low-frequency/high-frequency ratio; SpO_2_ = peripheral oxygen saturation. The full set of within-condition time-point comparisons is reported in Supplementary Tables S3 and S4. Missing data were handled with pairwise deletion.

## Data Availability

The data presented in this study are available on request from the corresponding author. The data are not publicly available due to privacy and ethical restrictions.

## Supplementary Materials

The following supporting information can be downloaded at: https://www.mdpi.com/article/10.3390/children13060771/s1, Figure S1: Frequency of the Contemporary Artists Reported by the Mothers; Table S1: Between-Condition Wilcoxon Signed-Rank Tests for the LF/HF Ratio (All Pairwise Comparisons); Table S2: Between-Condition Wilcoxon Signed-Rank Tests for SpO_2_ (All Pairwise Comparisons); Table S3: Within-Condition Wilcoxon Signed-Rank Tests for SpO_2_ (All Time-Point Comparisons); Table S4: Within-Condition Wilcoxon Signed-Rank Tests for the LF/HF Ratio (All Time-Point Comparisons); Table S5: Artists and Composers Reported by the Mothers in the Prenatal Music Questionnaire; Table S6: Musical Characteristics of the Eight Songs Used in the Three Playlists.

## Abbreviations

The following abbreviations are used in this manuscript:

HF	High frequency
HRV	Heart Rate Variability
LF	Low frequency
LF/HF	Low-frequency/high-frequency ratio
NICU	Neonatal Intensive Care Unit
SpO_2_	Peripheral oxygen saturation

## Appendix A

### Appendix A.1

Music therapy questionnaires for parents: music listened to during pregnancy for pre-recorded music in the incubator.

1. What is your favorite music genre?
  - POP
  - POP ROCK
  - POP RAP
  - CLASSIC
  - Other
2. Did you listen to music for your baby during pregnancy? (Yes/no)
3. Did you listen to classical music for your baby during pregnancy? (Yes/no)
4. Which classical music composer did you listen to during your pregnancy? Please write the name of one composer that you listened to more.
5. Please write n. Three classical pieces for the composer you listened to for your baby during pregnancy.
6. Please write n. Four modern composers that you listened to more for your baby during pregnancy.
7. Please write n. Three songs for each composer you listened to for your baby during pregnancy.
8. Would you like to use prerecorded music for your baby inside the incubator? (Yes/no)
9. Would you like to sing and record for your baby in the incubator? (Yes/no)
10. Would you like to play an instrumental live for your baby in the incubator? (Yes/no)

### Appendix A.2

**Table A1:** Musical characteristics of the three repertoires: Jovanotti (Soft Pop), Vasco Rossi (Soft Rock), and W. A. Mozart (Classical).

	Tempo	Song Structure	Voice	Instruments	Songs
Jovanotti Soft pop	Adagio-Allegretto Sustained regular. Repetitive. Same time. No variations in the refrain.	(A-B-A): harmonic range I-IV-V-I, intro, verse, bridge, chorus equal harmonically.	Voice, 1 octave baritone.	Male voice. Electric bass. Electric and acoustic guitar. Piano. Drums and percussion. Accordion. Live string orchestra.	Per te—key C major; Allegretto; tempo 4/4; 5 min. A te—key C major; Adagio; tempo 4/4; 5 min. Baciami ancora—key E-flat major; Allegretto; tempo 4/4; 5 min. Acoustic version. Album “Back Up”.
Vasco Rossi Soft rock	Adagio-Allegretto Lively. Regular in the verse. Change in tempo and rhythmic-harmonic variation in the refrain.	(A-A-B-A-C-A): intro, verse, bridge. Refrain rhythmic–harmonic variation. Melody and countermelody.	Voice 2-octave baritone.	Male voice. Piano. Harp. Drums. Chorus. Timpani. Keyboard and synthesizer. Percussion. Symphony orchestra.	Alba Chiara—key C major; Allegretto; tempo 4/4; 5 min. Anima fragile—key D major; Adagio; tempo 4/4; 5 min. Una canzone per te—key C major; Adagio; tempo 4/4; 5 min. Teatro la Scala string orchestra. Album “L’altra metà del cielo”.
W. A. Mozart Classical	Andantino Adagio	Andantino, KV 299. Form (A-B-C-D-C-B-A). Repetitive. Harp and flute solo. Adagio, KV 622. Form (A-B-A’). Repetitive. Clarinet solo.	No voice. Symphony orchestra.	Symphony orchestra, flute, harp, 2 oboes, 2 horns, strings. Harp. Flute. Symphony orchestra, flutes, bassoons, horns, strings. Clarinet solo.	Mozart KV 299 (movt. 2)—key F major; Andantino; tempo 3/4; 7 min 30 s’. Mozart KV 622 (movt. 2)—key D major, Adagio; tempo 3/4; 7 min 30 s. Maestro Hebert von Karajan (conductor). Berliner Philharmoniker Orchestra
