# Supplementary material for: Effects of Music Genres Reflecting Maternal Listening Preferences During Pregnancy on Distress Markers in Italian Preterm Infants
Source: Children (Basel). 2026 Jun 2;13(6):771. doi: 10.3390/children13060771 (PMC13298264; doi:10.3390/children13060771)
Supplement: Supplementary file 1 [file children-13-00771-s001.zip › children-4320035-supplementary.pdf]

Supplementary Materials

Table S1. Between-condition Wilcoxon signed-rank tests for the LF/HF ratio (all pairwise comparisons)

Comparison	*Z*	*p*	p(FDR)	*r*
Pre-test				
Jovanotti vs Control	-0.80	.426	.776	.17
Vasco Rossi vs Control	-1.38	.168	.776	.29
Mozart vs Control	-1.73	.083	.208	.36
Vasco Rossi vs Jovanotti	-2.38	.017	.135	.48
Mozart vs Jovanotti	-2.38	.018	.135	.47
Mozart vs Vasco Rossi	-0.36	.716	.940	.07
Phase 1				
Jovanotti vs Control	-0.61	.543	.776	.13
Vasco Rossi vs Control	-0.82	.412	.776	.17
Mozart vs Control	-1.09	.278	.463	.22
Vasco Rossi vs Jovanotti	-0.55	.581	.940	.11
Mozart vs Jovanotti	-0.55	.585	.940	.11
Mozart vs Vasco Rossi	-0.11	.914	.940	.02
Phase 2				
Jovanotti vs Control	-0.70	.484	.776	.15
Vasco Rossi vs Control	-1.52	.128	.776	.32
Mozart vs Control	-0.46	.648	.783	.09
Vasco Rossi vs Jovanotti	-0.63	.527	.940	.13
Mozart vs Jovanotti	-0.37	.713	.940	.07
Mozart vs Vasco Rossi	-0.07	.940	.940	.01
Phase 3				
Jovanotti vs Control	-0.43	.664	.830	.09
Vasco Rossi vs Control	-0.30	.768	.853	.07
Mozart vs Control	-0.28	.783	.783	.06
Vasco Rossi vs Jovanotti	-0.18	.855	.940	.04
Mozart vs Jovanotti	-0.30	.764	.940	.06

Mozart vs Vasco Rossi	-0.74	.458	.940	.15
Post-test				
Jovanotti vs Control	-0.03	.976	.976	.01
Vasco Rossi vs Control	-1.10	.274	.776	.23
Mozart vs Control	-1.80	.072	.208	.37
Vasco Rossi vs Jovanotti	-1.47	.143	.715	.29
Mozart vs Jovanotti	-1.08	.280	.840	.21
Mozart vs Vasco Rossi	-1.09	.276	.840	.22

Note. Full set of between-condition comparisons for the LF/HF ratio, by time point. Z = test statistic; p = uncorrected significance; p(FDR) = significance after Benjamini-Hochberg false discovery rate correction; r = effect size, computed as r = |Z|/√N. For each music condition compared with the no-music (control) condition, the correction was applied within the corresponding pre-specified hypothesis family; genre-to-genre comparisons were corrected within a separate family for each outcome. Following Cohen, r ≈ .10, .30, and .50 indicate small, medium, and large effects. Values of p(FDR) < .05 are shown in bold.

Table S2. Between-condition Wilcoxon signed-rank tests for SpO_2 (all pairwise comparisons)

**Comparison**	*Z*	*p*	**p(FDR)**	*r*
Pre-test				
Jovanotti vs Control	-0.20	.843	.958	.04
Vasco Rossi vs Control	-1.45	.146	.415	.29
Mozart vs Control	-0.59	.558	.693	.11
Vasco Rossi vs Jovanotti	-1.83	.067	.168	.37
Mozart vs Jovanotti	-0.66	.509	.694	.13
Mozart vs Vasco Rossi	-0.57	.572	.707	.11
Phase 1				
Jovanotti vs Control	-0.05	.958	.958	.01
Vasco Rossi vs Control	-1.39	.166	.415	.28
Mozart vs Control	-0.40	.693	.693	.08
Vasco Rossi vs Jovanotti	-1.25	.211	.396	.25
Mozart vs Jovanotti	-0.51	.613	.707	.10
Mozart vs Vasco Rossi	-1.10	.270	.450	.22
Phase 2				
Jovanotti vs Control	-0.07	.943	.958	.01
Vasco Rossi vs Control	-0.17	.863	.958	.03
Mozart vs Control	-2.31	.021	**.035**	.45
Vasco Rossi vs Jovanotti	-0.26	.792	.829	.05
Mozart vs Jovanotti	-2.03	.043	.168	.41
Mozart vs Vasco Rossi	-2.14	.032	.168	.43
Phase 3				
Jovanotti vs Control	-1.86	.063	.415	.37
Vasco Rossi vs Control	-1.52	.128	.415	.30
Mozart vs Control	-3.11	.002	**.010**	.60
Vasco Rossi vs Jovanotti	-0.72	.473	.694	.14
Mozart vs Jovanotti	-1.37	.171	.366	.27
Mozart vs Vasco Rossi	-1.89	.059	.168	.38
Post-test				
Jovanotti vs Control	-0.36	716	.958	.07

Vasco Rossi vs Control	-0.67	.501	.958	.13
Mozart vs Control	-2.73	.006	**.015**	.53
Vasco Rossi vs Jovanotti	-0.22	.829	.829	.04
Mozart vs Jovanotti	-1.84	.065	.168	.37
Mozart vs Vasco Rossi	-1.94	.052	.168	.39

Note. Full set of between-condition comparisons for SpO_2, by time point. Z = test statistic; p = uncorrected significance; p(FDR) = significance after Benjamini-Hochberg false discovery rate correction; r = effect size, computed as $r = |Z|/\sqrt{N}$. For each music condition compared with the no-music (control) condition, the correction was applied within the corresponding pre-specified hypothesis family; genre-to-genre comparisons were corrected within a separate family for each outcome. Following Cohen, r ≈ .10, .30, and .50 indicate small, medium, and large effects. Values of p(FDR) < .05 are shown in bold.

Table S3. Within-condition Wilcoxon signed-rank tests for SpO_2 (all time-point comparisons)

**Comparison**	***Z***	***p***	**p(FDR)**	***r***
Control				
Phase 1 vs Pre-test	-0.50	.618	.696	.10
Phase 2 vs Pre-test	-1.35	.178	.580	.26
Phase 3 vs Pre-test	-0.72	.475	.696	.14
Post-test vs Pre-test	-0.46	.647	.696	.09
Phase 2 vs Phase 1	-1.83	.067	.580	.35
Phase 3 vs Phase 1	-0.81	.418	.696	.16
Post-test vs Phase 1	-0.39	.696	.696	.07
Phase 3 vs Phase 2	-1.20	.232	.580	.23
Post-test vs Phase 2	-1.35	.177	.580	.26
Phase 3 vs Post-test	-0.66	.513	.696	.13
Jovanotti				
Phase 1 vs Pre-test	-0.34	.737	.819	.07
Phase 2 vs Pre-test	-2.13	.033	.082	.43
Phase 3 vs Pre-test	-3.11	.002	**.020**	.62
Post-test vs Pre-test	-0.14	.891	.891	.03
Phase 2 vs Phase 1	-1.86	.063	.105	.37
Phase 3 vs Phase 1	-2.18	.029	.082	.44
Post-test vs Phase 1	-0.37	.708	.819	.07
Phase 3 vs Phase 2	-1.92	.055	.105	.38
Post-test vs Phase 2	-0.67	.503	.719	.13
Phase 3 vs Post-test	-2.69	.007	**.035**	.54
Vasco Rossi				
Phase 1 vs Pre-test	-1.06	.290	.488	.21
Phase 2 vs Pre-test	-0.15	.882	.896	.03
Phase 3 vs Pre-test	-1.12	.263	.488	.22
Post-test vs Pre-test	-0.82	.414	.591	.16
Phase 2 vs Phase 1	-0.13	.896	.896	.03
Phase 3 vs Phase 1	-0.57	.568	.710	.11
Post-test vs Phase 1	-1.53	.126	.488	.31

Phase 3 vs Phase 2	-1.05	.293	.488	.21
Post-test vs Phase 2	-1.17	.241	.488	.23
Phase 3 vs Post-test	-2.42	.015	.150	.48
Mozart				
Phase 1 vs Pre-test	-0.08	.939	.939	.01
Phase 2 vs Pre-test	-3.89	<.001	**.001**	.75
Phase 3 vs Pre-test	-3.71	<.001	**.001**	.71
Post-test vs Pre-test	-2.70	.007	**.012**	.52
Phase 2 vs Phase 1	-3.62	<.001	**.001**	.70
Phase 3 vs Phase 1	-3.56	<.001	**.001**	.69
Post-test vs Phase 1	-2.79	.005	**.010**	.54
Phase 3 vs Phase 2	-1.02	.309	.343	.20
Post-test vs Phase 2	-1.85	.064	.080	.36
Phase 3 vs Post-test	-2.15	.032	**.046**	.41

Note. Full set of within-condition time-point comparisons for SpO_2, by condition. Z = test statistic; p = uncorrected significance; p(FDR) = significance after Benjamini-Hochberg false discovery rate correction, applied within each condition-by-outcome family; r = effect size, computed as $r = |Z|/\sqrt{N}$. Following Cohen, r ≈ .10, .30, and .50 indicate small, medium, and large effects. Values of p(FDR) < .05 are shown in bold. Missing data were handled with pairwise deletion.

Table S4. Within-condition Wilcoxon signed-rank tests for the LF/HF ratio (all time-point comparisons)

**Comparison**	***Z***	***p***	**p(FDR)**	***r***
Control				
Phase 1 vs Pre-test	-0.43	.670	.838	.09
Phase 2 vs Pre-test	-0.55	.584	.834	.12
Phase 3 vs Pre-test	-0.93	.355	.775	.20
Post-test vs Pre-test	-0.73	.465	.775	.15
Phase 2 vs Phase 1	-0.17	.864	.864	.04
Phase 3 vs Phase 1	-0.76	.447	.775	.16
Post-test vs Phase 1	-0.29	.775	.861	.06
Phase 3 vs Phase 2	-1.71	.242	.775	.36
Post-test vs Phase 2	-0.77	.440	.775	.16
Phase 3 vs Post-test	-0.79	.429	.775	.17
Jovanotti				
Phase 1 vs Pre-test	-1.88	.060	.200	.37
Phase 2 vs Pre-test	-2.02	.043	.200	.40
Phase 3 vs Pre-test	-1.90	.058	.200	.38
Post-test vs Pre-test	-0.72	.469	.938	.14
Phase 2 vs Phase 1	-0.37	.713	.989	.07
Phase 3 vs Phase 1	-0.01	.989	.989	.00
Post-test vs Phase 1	-1.33	.182	.455	.27
Phase 3 vs Phase 2	-0.31	.757	.989	.06
Post-test vs Phase 2	-0.12	.903	.989	.03
Phase 3 vs Post-test	-0.21	.833	.989	.04
Vasco Rossi				
Phase 1 vs Pre-test	-0.26	.798	.841	.05
Phase 2 vs Pre-test	-0.34	.737	.841	.07
Phase 3 vs Pre-test	-0.71	.475	.841	.14
Post-test vs Pre-test	-0.35	.726	.841	.07
Phase 2 vs Phase 1	-0.67	.501	.841	.13
Phase 3 vs Phase 1	-0.20	.841	.841	.04
Post-test vs Phase 1	-0.20	.840	.841	.04

Phase 3 vs Phase 2	-0.71	.475	.841	.14
Post-test vs Phase 2	-0.20	.840	.841	.04
Phase 3 vs Post-test	-0.97	.331	.841	.20
Mozart				
Phase 1 vs Pre-test	-0.79	.428	.869	.15
Phase 2 vs Pre-test	-1.01	.313	.869	.19
Phase 3 vs Pre-test	-0.37	.713	.869	.07
Post-test vs Pre-test	-0.17	.869	.869	.03
Phase 2 vs Phase 1	-0.29	.773	.869	.06
Phase 3 vs Phase 1	-0.46	.648	.869	.09
Post-test vs Phase 1	-1.66	.097	.869	.32
Phase 3 vs Phase 2	-0.76	.446	.869	.15
Post-test vs Phase 2	-1.20	.230	.869	.23
Phase 3 vs Post-test	-0.18	.859	.869	.04

Note. Full set of within-condition time-point comparisons for the LF/HF ratio, by condition. Z = test statistic; p = uncorrected significance; p(FDR) = significance after Benjamini-Hochberg false discovery rate correction, applied within each condition-by-outcome family; r = effect size, computed as $r = |Z|/\sqrt{N}$. Following Cohen, r ≈ .10, .30, and .50 indicate small, medium, and large effects. Values of p(FDR) < .05 are shown in bold. Missing data were handled with pairwise deletion.

Table S5. Artists and composers reported by the mothers in the prenatal music questionnaire

**Artist/composer**	***n***
Contemporary artists	
Jovanotti	27
Vasco Rossi	26
Elisa	11
Gianna Nannini	5
J-Ax	4
Giorgia	3
Fedez	2
Queen	2
Metallica	2
Måneskin	2
Other artists (one mention each)	16
Classical composers	
Mozart	19
Other composers (one mention each)	5

Note. Counts indicate the number of mothers among the 30 surveyed who reported each artist or composer in the open-ended questionnaire items. Several mothers reported more than one artist, so counts exceed the number of respondents. Three mothers reported no contemporary artist; six reported no classical composer. Other contemporary artists reported once: Brunori Sas, Vinicio Capossela, Adriano Celentano, Franco Battiato, Laura Pausini, Lucio Battisti, Alvaro Soler, Francesca Michielin, Eros Ramazzotti, Whitney Houston, Mina, Paola e Chiara, Claudio Baglioni, 883, Davena, and the Beatles. Other classical composers reported once: Chopin, Beethoven, Vivaldi, Morricone, and Einaudi. On this basis, Jovanotti, Vasco Rossi, and Mozart were selected to build the three playlists.

Figure S1. Frequency of the contemporary artists reported by the mothers

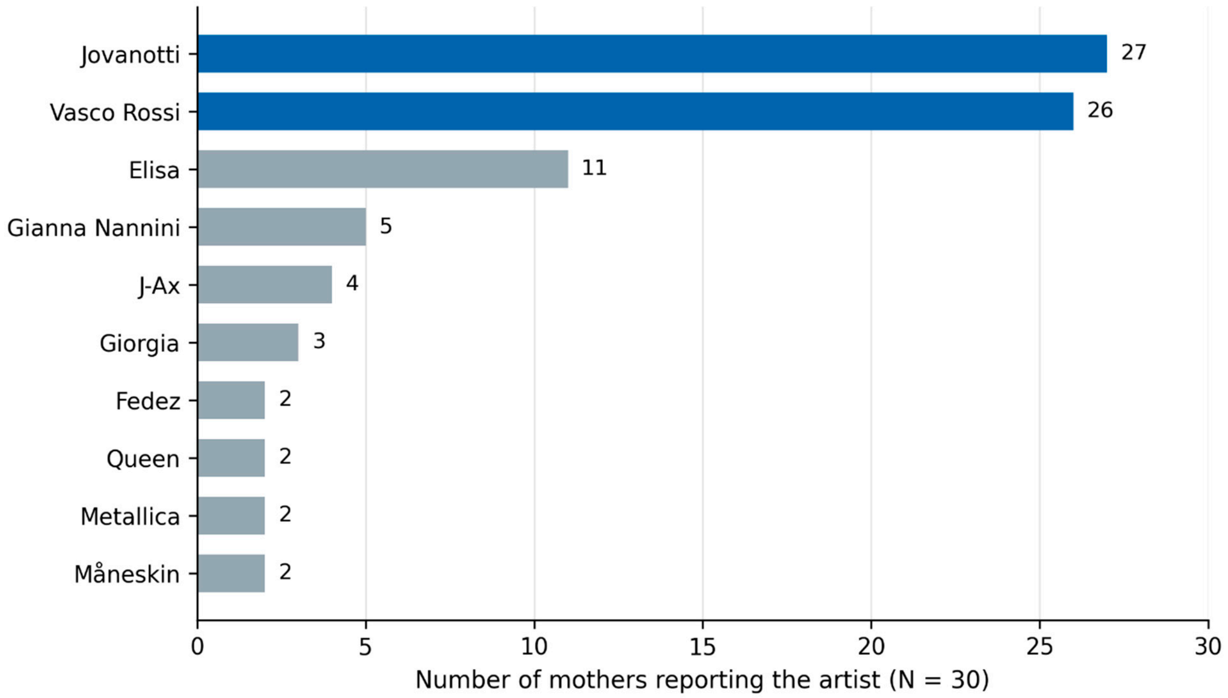

Note. The number of mothers (out of 30 surveyed) who reported on each contemporary artist. Only artists reported by at least two mothers are shown. Jovanotti and Vasco Rossi (highlighted) were the two most frequently reported artists and were selected for the soft-pop and soft-rock playlists.

Table S6. Musical characteristics of the eight songs used in the three playlists

Composer and song	Genre	Key	Tempo	Rhythm and structure	Voice (range)	Instrumentation	Lyrical theme
Mozart, Concerto KV 299 (movt. 2)	Classical	F major	Andantino	Slow and regular; bipartite instrumental form with flute and harp in dialogue and a recurring main theme.	Instrumental (flute, harp)	Symphony orchestra: flute, harp, 2 oboes, 2 horns, strings	Instrumental (no lyrics)
Mozart, Concerto KV 622 (movt. 2)	Classical	D major	Adagio	Slow and regular; simplified ternary form with transparent harmony (late-Mozart style).	Instrumental (clarinet)	Symphony orchestra: flutes, bassoons, horns, clarinet, strings	Instrumental (no lyrics)
Jovanotti, "A te"	Soft pop	C major	Adagio	Slow, regular, repetitive; canon over a I-IV-V-I progression with a recurring melody; harmonic range 1-4-5-1; intro; verse; bridge; chorus equal harmonically.	Male voice (about 1 octave)	Voice, electric bass, electric and acoustic guitar, piano, drums and percussion, accordion, live strings orchestra	Dedication to the artist's wife
Jovanotti, "Per te"	Soft pop	C major	Allegretto	Sustained, regular, repetitive; simple soft pop form (A-B-A) over a I-IV-V-I progression; harmonic range 1-4-5-1; intro; verse; bridge; chorus equal harmonically.	Male voice (about 1 octave)	Voice, electric bass, electric and acoustic guitar, piano, drums and percussion, accordion, live strings	Dedication to the artist's newborn daughter

Jovanotti, "Baciami ancora"	Soft pop	E-flat major	Allegretto	Sustained, regular, repetitive; simple pop form (A-B-A) over a I-IV-V-I progression; harmonic range 1-4-5-1; intro; verse; bridge; chorus equal harmonically.	Male voice (about 1 octave)	Voice, electric bass, electric and acoustic guitar, piano, drums and percussion, accordion, live strings	Film soundtrack
Vasco Rossi, "Alba chiara"	Soft rock	C major	Adagio to Allegro	Lively; regular in the verse, with a tempo change and rhythmic-harmonic variation in the refrain; contrasting rock form (A-A-B-A-C-A) with melody and counter-melody.	Male voice (about 2 octaves)	Symphonic orchestra, voice, piano, harp, drums, choir, timpani, percussion	Inspired by a teenage girl
Vasco Rossi, "Anima fragile"	Soft rock	D major	Adagio to Allegro	Lively; regular verse, tempo change and rhythmic-harmonic variation in the refrain; contrasting rock form (A-A-B-A-C-A).	Male voice (about 2 octaves)	Symphonic orchestra, voice, piano, harp, drums, choir, timpani, percussion	Dedication to the artist's late father
Vasco Rossi, "Una canzone per te"	Soft rock	C major	Adagio to Andantino	Andantino, regular in the verse, with rhythmic-harmonic variation in the refrain; contrasting rock form (A-A-B-A-C-A).	Male voice (about 2 octaves)	Voice, synthesizer, electric and 12-string guitar, bass, drums, choir, percussion, keyboard	Linked to the story of "Alba chiara"

Note. A certified music therapist conducted a structured analysis of the eight songs that comprise the three playlists. The detailed analysis is reported in Appendix 2.
